# (4-Nitrophenyl)(1,2,3,9-tetrahydro­pyrrolo[2,1-*b*]quinazolin-3-yl)methanol monohydrate

**DOI:** 10.1107/S1600536811021775

**Published:** 2011-06-18

**Authors:** Burkhon Zh. Elmuradov, Charoskhon E. Makhmadiyarova, Kambarali K. Turgunov, Bakhodir Tashkhodjaev, Khusnutdin M. Shakhidoyatov

**Affiliations:** a S.Yunusov Institute of the Chemistry of Plant Substances, Academy of Sciences of Uzbekistan, Mirzo Ulugbek Str., 77, Tashkent 100170, Uzbekistan

## Abstract

In the crystal structure of the title compound, C_18_H_17_N_3_O_3_·H_2_O, the mol­ecules are linked by O—H⋯O and O—H⋯N hydrogen bonds, resulting in a chain along the *a* axis. The crystal structure is stabilized by weak inter­molecular C—H⋯π (ring) hydrogen bonds and aromatic π⋯π stacking inter­actions [centroid–centroid distance = 3.902 (1) Å] between the pyrimidino rings of the quinazoline system. The tricyclic quinazoline fragment is almost planar (rms deviation = 0.0139 Å) with the two methylene C atoms of the pyrrolo ring deviating by 0.148 (2) and −0.081 (3) Å from the plane through the other atoms. The 4-nitrophenyl ring makes a dihedral angle of 12.55 (7)° with the tricyclic ring system.

## Related literature

For general background to tricyclic quinazoline alkaloids, see: Shakhidoyatov *et al.* (1988 ▶). For the synthesis of 1,2,3,9-tetra­hydro-pyrrolo­[2,1-*b*]quinazoline, see: Jahng *et al.* (2008 ▶). For the physiological activity of quinazoline derivatives, see: Al-Shamma *et al.* (1981 ▶); Yunusov *et al.* (1978 ▶).
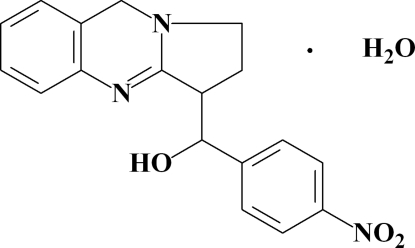


## Experimental

### 

#### Crystal data

C_18_H_17_N_3_O_3_·H_2_O
*M* *_r_* = 341.36
Triclinic, 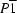

*a* = 6.2459 (7) Å
*b* = 11.4629 (11) Å
*c* = 11.8400 (13) Å
α = 91.932 (8)°
β = 95.589 (9)°
γ = 104.747 (9)°
*V* = 814.37 (15) Å^3^
*Z* = 2
Cu *K*α radiation
μ = 0.83 mm^−1^
*T* = 295 K
0.50 × 0.35 × 0.15 mm

#### Data collection

Oxford Diffraction Xcalibur Ruby diffractometer
Absorption correction: multi-scan (*CrysAlis PRO*; Oxford Diffraction, 2009 ▶) *T*_min_ = 0.793, *T*_max_ = 1.000
4649 measured reflections
2867 independent reflections
2076 reflections with *I* > 2σ(*I*)
*R*_int_ = 0.022

#### Refinement

*R*[*F* ^2^ > 2σ(*F* ^2^)] = 0.044
*wR*(*F* ^2^) = 0.124
*S* = 1.03
2867 reflections
238 parameters
H atoms treated by a mixture of independent and constrained refinement
Δρ_max_ = 0.18 e Å^−3^
Δρ_min_ = −0.20 e Å^−3^

### 

Data collection: *CrysAlis PRO* (Oxford Diffraction, 2009 ▶); cell refinement: *CrysAlis PRO*; data reduction: *CrysAlis PRO*; program(s) used to solve structure: *SHELXS97* (Sheldrick, 2008 ▶); program(s) used to refine structure: *SHELXL97* (Sheldrick, 2008 ▶); molecular graphics: *XP* in *SHELXTL* (Sheldrick, 2008 ▶); software used to prepare material for publication: *publCIF* (Westrip, 2010 ▶).

## Supplementary Material

Crystal structure: contains datablock(s) I, global. DOI: 10.1107/S1600536811021775/rk2278sup1.cif

Structure factors: contains datablock(s) I. DOI: 10.1107/S1600536811021775/rk2278Isup2.hkl

Supplementary material file. DOI: 10.1107/S1600536811021775/rk2278Isup3.cml

Additional supplementary materials:  crystallographic information; 3D view; checkCIF report

## Figures and Tables

**Table 1:** Hydrogen-bond geometry (Å, °) *Cg*1 and *Cg*2 are the centroids of the N1,C2,N3,C4,C4*A*,C8*A* and C4*A*,C5–C8,C8*A* rings, respectively.

*D*—H⋯*A*	*D*—H	H⋯*A*	*D*⋯*A*	*D*—H⋯*A*
O1*W*—H1*W*⋯N1	0.86 (2)	1.89 (3)	2.751 (2)	174 (2)
O1*W*—H2*W*⋯O1^i^	0.89 (3)	1.97 (3)	2.835 (2)	162 (2)
O1—H1⋯O1*W*^ii^	0.95 (4)	1.71 (3)	2.660 (2)	173 (3)
C4—H4*B*⋯*Cg*1^iii^	0.97	2.92	3.634 (2)	131
C11—H11*A*⋯*Cg*2^iii^	0.97	2.94	3.681 (2)	134

Symmetry codes: (i) 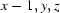; (ii) 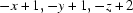; (iii) 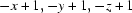.

**Fig. 1.**
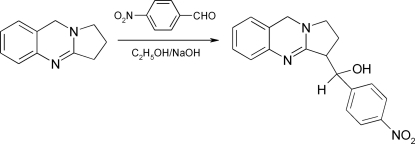
The reaction scheme.

**Fig. 2.**
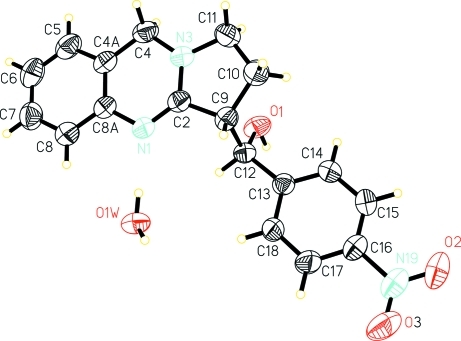
The molecular structure of title compound with atom labels. The displacement ellipsoidsand are drawn at 50% probability level. H atoms are presented as a small spheres of arbitrary radius.

**Fig. 3.**
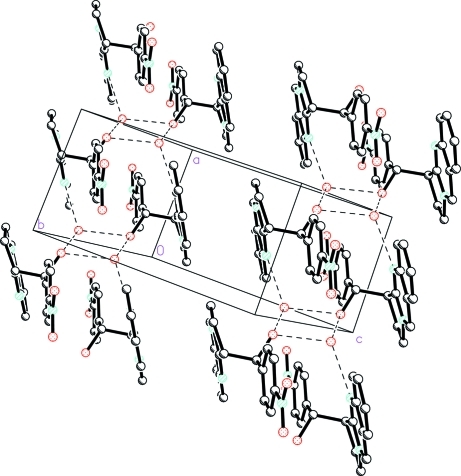
Packing diagramm, showing the formation of H-bonded (dashed lines) chains along [1 0 0]. H atoms are omitted for clarity.

## supplementary crystallographic information

### Comment

Tricyclic quinazoline alkaloids are a large group of heterocyclic compounds (Shakhidoyatov *et al.*, 1988; Jahng *et al.*, 2008). These compounds and their derivatives possess difference pharmacological activities (Al-Shamma *et al.*, 1981; Yunusov *et al.*, 1978). Reaction of 1,2,3,9-tetrahydro-pyrrolo[2,1-*b*]quinazoline with *p*-nitrobenzaldehyde in ethanol at present of sodium hydroxide leads to the formation of 3-(*p*-nitrophenyl)-hydroxymethyl-1,2,3,9-tetrahydro-pyrrolo[2,1- *b*]quinazoline (Fig. 1). The title molecule has two asymmetric centre. The crystal is a racemate of two optical antipodes. The asymmetric unit contains one molecule of 3-(*p*-nitrophenyl)-hydroxymethyl-1,2,3,9-tetrahydro-pyrrolo[2,1- *b*]quinazoline and one water molecule (Fig. 2). In the molecule tryciclic quinazoline fragment almost planar with of slightly twisting of atoms C9 and C10. Deviations of last atoms from plane of rest atoms (rms deviation = 0.0139Å) in the tricycle are 0.148 (2)Å and -0.081 (3)Å, respectively.

Hydroxyl groups of two centrosymmetrical related molecules of title compound and two water molecules form a H-bond regtangles (nearly). In addition the water molecules are hydrogen bonded to the title compound molecules through N1 atom (Table 1). In the result are formed H-bond chains along the *a* axis of the cell (Fig. 3). The observed structure is stabilized by weak C—H···π (Table 1) and aromatic π···π stacking interactions. A centrosymmetric π···π stacking interactions are observed between pyrimidino (N1/C2/N3/C4/C4A/C8A) rings of centrosymmetrically related molecules (*Cg*1···*Cg*1^i^ separation is 3.902 (1)Å, where symmetry code: (i) 1-*x*, 1-*y*, 1-*z*).

### Experimental

Sodium hydroxide (0.1 g, 2.5 mmol) was dissolved in 40 ml ethanol (80%), and 1,2,3,9-tetrahydro-pyrrolo[2,1-*b*]quinazoline hydrochloride (0.448 g, 2 mmol) and *p*-nitrobenzaldehyde (0.604 g, 4 mmol) were added (Fig. 1). Reaction mixture was left at 278 (1) K for 5 weeks. Light yellow crystals (m.p. 473–474 K) suitable for X-ray diffraction were isolated in 72% yield (0.44 g).

^1^H NMR (400 MHz, C_5_H_5_N): 8.1 (2*H*, d, J = 8.8, H-3',5'), 7.9 (1*H*, s, OH), 7.67 (1*H*, d, J = 8.6, H-8), 7.67 (2*H*, d, J = 8.6, H-2',6'), 7.14 (2*H*, t, J = 8.6, H-6), 6.9 (1*H*, td, J = 8.6, J = 2.0, H-7), 6.8 (1*H*, d, J = 7.6, H-5), 5.06 (1*H*, d, J = 8.4, CH), 4.17 (1*H*, s, 9-H), 2.94 (1*H*, q, J = 8.6, H-1a), 2.78 (1*H*, q, J = 9.4, H-1b), 2.65 (2*H*, td, J = 8.6, J = 3.3, 3-H), 1.52 (2*H*, m, 2-H).

Mass (m/*z*, %): 323 ([*M*]^+^, 5.6), 305 ([*M*-H_2_O]^+^, 6.3), 201 ([*M*-C_6_H_4_NO_2_]^+^, 2.8), 171 ([*M*-(HO)CHC_6_H_4_NO_2_]^+^, 100), 151 (51), 76 (55).

### Refinement

Carbon-bound H atoms were positioned geometrically and treated as riding on their C atoms, with C—H distances of 0.93Å (aromatic) and 0.97Å (methylene) and were refined with *U*_iso_(H) = 1.2*U*_eq_(C)]. The H atoms of hydroxyl group [O—H = 0.95 (3)Å] and the water molecule [O—H = 0.86 (3)Å and 0.89 (3)Å] involved in the intermolecular hydrogen bonds were located by difference Fourier map and refined freely.

### Crystal data

**Table:**

C_18_H_17_N_3_O_3_·H_2_O	*Z* = 2
*M**_r_* = 341.36	*F*(000) = 360
Triclinic, *P*1	*D*_x_ = 1.392 Mg m^−^^3^
Hall symbol: -P 1	Melting point = 473(2)–474(2) K
*a* = 6.2459 (7) Å	Cu *K*α radiation, λ = 1.54180 Å
*b* = 11.4629 (11) Å	Cell parameters from 1912 reflections
*c* = 11.8400 (13) Å	θ = 3.8–66.8°
α = 91.932 (8)°	µ = 0.83 mm^−^^1^
β = 95.589 (9)°	*T* = 295 K
γ = 104.747 (9)°	Prism, light-yellow
*V* = 814.37 (15) Å^3^	0.50 × 0.35 × 0.15 mm

### Data collection

**Table:**

Oxford Diffraction Xcalibur Ruby diffractometer	2867 independent reflections
Radiation source: Enhance (Cu) X-ray Source	2076 reflections with *I* > 2σ(*I*)
graphite	*R*_int_ = 0.022
Detector resolution: 10.2576 pixels mm^-1^	θ_max_ = 66.9°, θ_min_ = 3.8°
ω–scans	*h* = −7→7
Absorption correction: multi-scan (*CrysAlis PRO*; Oxford Diffraction, 2009)	*k* = −13→13
*T*_min_ = 0.793, *T*_max_ = 1.000	*l* = −11→14
4649 measured reflections	

### Refinement

**Table:**

Refinement on *F*^2^	Primary atom site location: structure-invariant direct methods
Least-squares matrix: full	Secondary atom site location: difference Fourier map
*R*[*F*^2^ > 2σ(*F*^2^)] = 0.044	Hydrogen site location: inferred from neighbouring sites
*wR*(*F*^2^) = 0.124	H atoms treated by a mixture of independent and constrained refinement
*S* = 1.03	*w* = 1/[σ^2^(*F*_o_^2^) + (0.0752*P*)^2^ + 0.0389*P*] where *P* = (*F*_o_^2^ + 2*F*_c_^2^)/3
2867 reflections	(Δ/σ)_max_ = 0.001
238 parameters	Δρ_max_ = 0.18 e Å^−^^3^
0 restraints	Δρ_min_ = −0.20 e Å^−^^3^

### Special details

**Table:**

Geometry. All s.u.'s (except the s.u. in the dihedral angle between two l.s. planes) are estimated using the full covariance matrix. The cell s.u.'s are taken into account individually in the estimation of s.u.'s in distances, angles and torsion angles; correlations between s.u.'s in cell parameters are only used when they are defined by crystal symmetry. An approximate (isotropic) treatment of cell s.u.'s is used for estimating s.u.'s involving l.s. planes.
Refinement. Refinement of *F*^2^ against ALL reflections. The weighted *R*-factor *wR* and goodness of fit *S* are based on *F*^2^, conventional *R*-factors *R* are based on *F*, with *F* set to zero for negative *F*^2^. The threshold expression of *F*^2^ > σ(*F*^2^) is used only for calculating *R*-factors(gt) *etc*. and is not relevant to the choice of reflections for refinement. *R*-factors based on *F*^2^ are statistically about twice as large as those based on *F*, and *R*-factors based on ALL data will be even larger.

### Fractional atomic coordinates and isotropic or equivalent isotropic displacement parameters (Å^2^)

**Table:**

	*x*	*y*	*z*	*U*_iso_*/*U*_eq_	
O1	0.8540 (2)	0.61715 (12)	0.92847 (12)	0.0555 (4)	
O2	0.6598 (3)	1.09493 (15)	1.24380 (17)	0.0991 (6)	
O3	0.3240 (3)	0.99048 (18)	1.24831 (17)	0.1005 (6)	
N1	0.3825 (2)	0.45278 (13)	0.71834 (12)	0.0471 (4)	
C2	0.5561 (3)	0.54174 (16)	0.71298 (14)	0.0427 (4)	
N3	0.7234 (2)	0.54380 (13)	0.65065 (13)	0.0487 (4)	
C4	0.7416 (3)	0.44111 (18)	0.58074 (16)	0.0537 (5)	
H4A	0.8783	0.4197	0.6060	0.064*	
H4B	0.7473	0.4624	0.5023	0.064*	
C4A	0.5454 (3)	0.33484 (17)	0.58861 (15)	0.0475 (4)	
C5	0.5291 (4)	0.22532 (19)	0.53039 (18)	0.0636 (6)	
H5A	0.6415	0.2178	0.4868	0.076*	
C6	0.3493 (4)	0.1273 (2)	0.5359 (2)	0.0700 (6)	
H6A	0.3414	0.0545	0.4964	0.084*	
C7	0.1821 (4)	0.13746 (18)	0.59957 (19)	0.0643 (6)	
H7A	0.0599	0.0719	0.6028	0.077*	
C8	0.1959 (3)	0.24550 (17)	0.65902 (17)	0.0558 (5)	
H8A	0.0828	0.2518	0.7027	0.067*	
C8A	0.3761 (3)	0.34467 (16)	0.65443 (14)	0.0451 (4)	
C9	0.5996 (3)	0.65999 (15)	0.78287 (15)	0.0457 (4)	
H9A	0.4710	0.6940	0.7698	0.055*	
C10	0.8016 (4)	0.74163 (18)	0.73443 (18)	0.0609 (5)	
H10A	0.7562	0.8017	0.6888	0.073*	
H10B	0.9129	0.7828	0.7955	0.073*	
C11	0.8949 (3)	0.65817 (18)	0.66131 (18)	0.0575 (5)	
H11A	0.9194	0.6898	0.5874	0.069*	
H11B	1.0345	0.6483	0.6980	0.069*	
C12	0.6388 (3)	0.63750 (15)	0.90930 (14)	0.0433 (4)	
H12A	0.5295	0.5631	0.9240	0.052*	
C13	0.6112 (3)	0.73811 (15)	0.98840 (14)	0.0425 (4)	
C14	0.7810 (3)	0.83964 (17)	1.02340 (17)	0.0563 (5)	
H14A	0.9196	0.8485	0.9973	0.068*	
C15	0.7481 (3)	0.92825 (17)	1.09669 (18)	0.0611 (5)	
H15A	0.8630	0.9967	1.1193	0.073*	
C16	0.5446 (3)	0.91394 (16)	1.13547 (16)	0.0510 (5)	
C17	0.3738 (3)	0.8124 (2)	1.10522 (19)	0.0652 (6)	
H17A	0.2373	0.8026	1.1340	0.078*	
C18	0.4091 (3)	0.72560 (19)	1.03133 (19)	0.0619 (6)	
H18A	0.2942	0.6568	1.0098	0.074*	
N19	0.5065 (4)	1.00649 (17)	1.21455 (16)	0.0689 (5)	
H1	0.878 (4)	0.600 (3)	1.006 (3)	0.113 (10)*	
O1W	0.0490 (2)	0.43285 (14)	0.85779 (12)	0.0573 (4)	
H1W	0.151 (4)	0.444 (2)	0.812 (2)	0.081 (8)*	
H2W	−0.024 (4)	0.490 (3)	0.864 (2)	0.107 (10)*	

### Atomic displacement parameters (Å^2^)

**Table:**

	*U*^11^	*U*^22^	*U*^33^	*U*^12^	*U*^13^	*U*^23^
O1	0.0543 (8)	0.0736 (9)	0.0481 (8)	0.0331 (7)	0.0083 (6)	0.0005 (7)
O2	0.1175 (15)	0.0596 (10)	0.1127 (15)	0.0132 (10)	0.0153 (11)	−0.0336 (10)
O3	0.0964 (13)	0.1053 (13)	0.1106 (15)	0.0431 (11)	0.0318 (11)	−0.0328 (11)
N1	0.0458 (8)	0.0505 (9)	0.0447 (8)	0.0111 (7)	0.0108 (7)	−0.0062 (7)
C2	0.0426 (9)	0.0493 (10)	0.0379 (9)	0.0156 (8)	0.0039 (7)	0.0014 (7)
N3	0.0474 (8)	0.0537 (9)	0.0458 (9)	0.0118 (7)	0.0136 (7)	−0.0008 (7)
C4	0.0515 (11)	0.0680 (12)	0.0460 (11)	0.0219 (9)	0.0116 (8)	−0.0019 (9)
C4A	0.0520 (10)	0.0563 (11)	0.0376 (9)	0.0212 (9)	0.0036 (8)	−0.0013 (8)
C5	0.0705 (14)	0.0696 (14)	0.0563 (12)	0.0293 (11)	0.0087 (10)	−0.0112 (10)
C6	0.0858 (16)	0.0566 (12)	0.0676 (14)	0.0239 (12)	0.0001 (12)	−0.0163 (11)
C7	0.0711 (14)	0.0504 (11)	0.0652 (13)	0.0071 (10)	0.0025 (11)	−0.0043 (10)
C8	0.0588 (12)	0.0556 (11)	0.0521 (12)	0.0125 (9)	0.0103 (9)	−0.0020 (9)
C8A	0.0498 (10)	0.0481 (10)	0.0381 (9)	0.0147 (8)	0.0038 (8)	0.0000 (7)
C9	0.0472 (10)	0.0466 (9)	0.0443 (10)	0.0153 (8)	0.0032 (8)	−0.0012 (8)
C10	0.0749 (14)	0.0518 (11)	0.0520 (12)	0.0069 (10)	0.0126 (10)	0.0050 (9)
C11	0.0522 (11)	0.0611 (12)	0.0553 (12)	0.0051 (9)	0.0111 (9)	0.0072 (9)
C12	0.0404 (9)	0.0465 (9)	0.0447 (10)	0.0130 (8)	0.0097 (7)	−0.0007 (8)
C13	0.0394 (9)	0.0471 (9)	0.0412 (9)	0.0121 (8)	0.0042 (7)	0.0001 (7)
C14	0.0465 (10)	0.0555 (11)	0.0630 (13)	0.0028 (9)	0.0182 (9)	−0.0053 (9)
C15	0.0610 (12)	0.0463 (10)	0.0670 (13)	−0.0034 (9)	0.0132 (10)	−0.0087 (9)
C16	0.0609 (12)	0.0478 (10)	0.0474 (10)	0.0211 (9)	0.0050 (9)	−0.0035 (8)
C17	0.0436 (11)	0.0751 (14)	0.0766 (14)	0.0156 (10)	0.0139 (10)	−0.0211 (11)
C18	0.0382 (10)	0.0673 (12)	0.0728 (14)	0.0025 (9)	0.0107 (9)	−0.0252 (10)
N19	0.0874 (14)	0.0598 (11)	0.0644 (12)	0.0304 (11)	0.0071 (10)	−0.0099 (9)
O1W	0.0554 (9)	0.0704 (9)	0.0539 (8)	0.0256 (7)	0.0187 (7)	0.0049 (7)

### Geometric parameters (Å, °)

**Table:**

O1—C12	1.420 (2)	C9—C12	1.534 (2)
O1—H1	0.95 (3)	C9—C10	1.540 (3)
O2—N19	1.217 (2)	C9—H9A	0.9800
O3—N19	1.216 (2)	C10—C11	1.527 (3)
N1—C2	1.294 (2)	C10—H10A	0.9700
N1—C8A	1.420 (2)	C10—H10B	0.9700
C2—N3	1.333 (2)	C11—H11A	0.9700
C2—C9	1.513 (2)	C11—H11B	0.9700
N3—C4	1.451 (2)	C12—C13	1.515 (2)
N3—C11	1.459 (2)	C12—H12A	0.9800
C4—C4A	1.505 (3)	C13—C14	1.380 (2)
C4—H4A	0.9700	C13—C18	1.382 (2)
C4—H4B	0.9700	C14—C15	1.381 (3)
C4A—C5	1.387 (3)	C14—H14A	0.9300
C4A—C8A	1.398 (2)	C15—C16	1.366 (3)
C5—C6	1.380 (3)	C15—H15A	0.9300
C5—H5A	0.9300	C16—C17	1.374 (3)
C6—C7	1.372 (3)	C16—N19	1.471 (2)
C6—H6A	0.9300	C17—C18	1.377 (3)
C7—C8	1.383 (3)	C17—H17A	0.9300
C7—H7A	0.9300	C18—H18A	0.9300
C8—C8A	1.388 (3)	O1W—H1W	0.86 (3)
C8—H8A	0.9300	O1W—H2W	0.89 (3)
			
C12—O1—H1	108.1 (17)	C11—C10—H10A	110.5
C2—N1—C8A	115.94 (14)	C9—C10—H10A	110.5
N1—C2—N3	126.93 (16)	C11—C10—H10B	110.5
N1—C2—C9	123.07 (15)	C9—C10—H10B	110.5
N3—C2—C9	109.97 (15)	H10A—C10—H10B	108.7
C2—N3—C4	123.89 (15)	N3—C11—C10	104.22 (14)
C2—N3—C11	114.25 (15)	N3—C11—H11A	110.9
C4—N3—C11	121.82 (14)	C10—C11—H11A	110.9
N3—C4—C4A	110.24 (14)	N3—C11—H11B	110.9
N3—C4—H4A	109.6	C10—C11—H11B	110.9
C4A—C4—H4A	109.6	H11A—C11—H11B	108.9
N3—C4—H4B	109.6	O1—C12—C13	112.19 (14)
C4A—C4—H4B	109.6	O1—C12—C9	107.19 (13)
H4A—C4—H4B	108.1	C13—C12—C9	113.63 (14)
C5—C4A—C8A	118.79 (18)	O1—C12—H12A	107.9
C5—C4A—C4	120.53 (17)	C13—C12—H12A	107.9
C8A—C4A—C4	120.68 (16)	C9—C12—H12A	107.9
C6—C5—C4A	121.3 (2)	C14—C13—C18	118.21 (17)
C6—C5—H5A	119.4	C14—C13—C12	123.21 (15)
C4A—C5—H5A	119.4	C18—C13—C12	118.53 (16)
C7—C6—C5	119.88 (19)	C13—C14—C15	120.99 (17)
C7—C6—H6A	120.1	C13—C14—H14A	119.5
C5—C6—H6A	120.1	C15—C14—H14A	119.5
C6—C7—C8	119.8 (2)	C16—C15—C14	119.12 (18)
C6—C7—H7A	120.1	C16—C15—H15A	120.4
C8—C7—H7A	120.1	C14—C15—H15A	120.4
C7—C8—C8A	120.87 (19)	C15—C16—C17	121.54 (17)
C7—C8—H8A	119.6	C15—C16—N19	120.08 (18)
C8A—C8—H8A	119.6	C17—C16—N19	118.35 (18)
C8—C8A—C4A	119.37 (17)	C16—C17—C18	118.45 (18)
C8—C8A—N1	118.37 (16)	C16—C17—H17A	120.8
C4A—C8A—N1	122.25 (16)	C18—C17—H17A	120.8
C2—C9—C12	109.34 (14)	C17—C18—C13	121.65 (18)
C2—C9—C10	103.65 (14)	C17—C18—H18A	119.2
C12—C9—C10	114.74 (15)	C13—C18—H18A	119.2
C2—C9—H9A	109.6	O3—N19—O2	123.18 (19)
C12—C9—H9A	109.6	O3—N19—C16	118.45 (19)
C10—C9—H9A	109.6	O2—N19—C16	118.4 (2)
C11—C10—C9	106.12 (15)	H1W—O1W—H2W	118 (3)

### Hydrogen-bond geometry (Å, °)

**Table:**

Cg1 and Cg2 are the centroids of the N1,C2,N3,C4,C4A,C8A and C4A,C5–C8,C8A rings, respectively.

**Table:**

*D*—H···*A*	*D*—H	H···*A*	*D*···*A*	*D*—H···*A*
O1W—H1W···N1	0.86 (2)	1.89 (3)	2.751 (2)	174 (2)
O1W—H2W···O1^i^	0.89 (3)	1.97 (3)	2.835 (2)	162 (2)
O1—H1···O1W^ii^	0.95 (4)	1.71 (3)	2.660 (2)	173 (3)
C4—H4B···Cg1^iii^	0.97	2.92	3.634 (2)	131
C11—H11A···Cg2^iii^	0.97	2.94	3.681 (2)	134

Symmetry codes:  (i) *x*−1, *y*, *z*; (ii) −*x*+1, −*y*+1, −*z*+2; (iii) −*x*+1, −*y*+1, −*z*+1.
